# Amelioration of Hepatic Encephalopathy Using *Dunaliella salina* Microalgae in Rats: Modulation of Hyperammonemia/TLR4

**DOI:** 10.1155/2021/8843218

**Published:** 2021-03-28

**Authors:** Farouk K. El-Baz, Rania Elgohary, Abeer Salama

**Affiliations:** ^1^Plant Biochemistry Department, National Research Centre (NRC), 33 El Buhouth St., Dokki, Cairo 12622, Egypt; ^2^Narcotics, Ergogenics and Poisons Department, National Research Centre (NRC), 33 El Buhouth St., Dokki, Cairo 12622, Egypt; ^3^Pharmacology Department, National Research Centre (NRC), 33 El Buhouth St., Dokki, Cairo 12622, Egypt

## Abstract

Hepatic encephalopathy (HE) is a neuropsychiatric disease that is developed as a complication of both acute and chronic liver failure affecting psychomotor dysfunction, memory, and concentration. This study is aimed at evaluating the therapeutic effects of *Dunaliella salina* (*D*. *salina*) microalgae in thioacetamide- (TAA-) induced HE in rats. HE was induced by TAA (200 mg/kg; i.p.) for three successive days. Forty male Wister albino rats were divided into 4 groups; the first group was served as a normal, and the second group was injected with TAA and served as TAA control. The third and fourth groups were administered *D*. *salina* (100 and 200 mg/kg; p.o.), respectively, after TAA injection for 7 days. The behavioral and biochemical markers as well as histological aspects of HE were estimated. This study revealed that TAA caused behavioral changes, oxidative stress, neuroinflammation, nuclear pyknosis, and neurons degeneration. *D. salina* improved liver function and decreased oxidative stress and inflammatory mediator as TLR4 protein expression. Also, *D*. *salina* elevated HSP-25 and IGF-1 as well as improved brain histopathological alterations. In conclusion, *D*. *salina* exerted a therapeutic potential against HE via its antioxidant, antiinflammatory and cytoprotective effects.

## 1. Introduction

Hepatic encephalopathy (HE) reflects a spectrum of neuropsychiatric abnormalities such as sensory abnormalities, psychomotor dysfunction, impaired memory, poor concentration, and increased reaction time. These manifestations are due to acute or chronic liver failure [1]. HE affects 45-70% of patients with liver cirrhosis and 10-20% of patients with Transjugular Porto Systemic Shunts, in developed countries [2]. HE causes hospitalization and high rates of mortality as a result of end-stage liver disease, reaching to coma [3].

In HE, impaired liver function elevates the levels of ammonia in the blood that crosses the blood-brain barrier and metabolized in the CNS [4]. In brain astrocytes, the ammonia detoxification is due to its incorporation into glutamine ultimately resulting in increased water entry and osmotic forces, astrocytes swelling, and cytotoxic edema [5]. Hyperammonemia induced inflammation that is not only a partner aggravating the hepatic diseases, but also exacerbates the neuropsychological disturbances in HE [6]. There is a cross talk between inflammatory mediators and ammonia in HE patients [7] such as toll-like receptors (TLRs), in acute or chronic hepatic diseases, that trigger inflammation [8] and act as critical determinants of HE severity via releasing proinflammatory mediators [9]. Moreover, TLR4 activation mediates oxidative stress in neurons and hepatocytes [10].

During oxidative stress, in HE patients, heat shock proteins (HSPs) (HSP-25 in rats and HSP27 in humans) have a role as an antioxidant, reducing reactive oxygen species (ROS) and elevating the intracellular glutathione [11]. Also, insulin-like growth factor-1 (IGF-1) is an endocrine *growth factor* produced in the liver for hepatocytes' growth, differentiation, and proliferation. IGF acts as a hepatoprotective against oxidative stress [12]. It improved liver function, suppressed oxidative liver damage, and increased antioxidative enzymes such as catalase, superoxide dismutase, and glutathione peroxidase, in fibrosis and liver cirrhosis rat model [13].

Thioacetamide (TAA), a hepatotoxin, used to induce hepatic failure and HE in experimental studies due to its efficacy in causing liver and brain disorders similar to human progressive hepatic disorders with brain involvement [14]. TAA undergoes metabolism by the cytochrome P450-producing metabolite which in turn responsible for hyperammonemia, oxidative stress, and hepatic necrosis [15].

Previous studies have proven the antioxidant and anti-inflammatory activities of different microalgal extracts [16]. *Dunaliella salina* (*D. salina)* microalgae are unicellular marine phytoplankton that belongs to the phylum Chlorophyta and family Dunaliellace [17] which contains large amounts of carotenoids at the stationary growth phase and has antioxidant and anticancer activity [18]. It has been shown that *D*. *salina* carotenoids can preserve hepatic enzyme activity as peroxidase, catalase, and superoxide dismutase which are involved in scavenging ROS [19]. Carotenoids have medicinal properties that are used in several diseases, such as cancer and diabetes, food supplements, cosmetics, and pharmaceuticals. Microalgae therapeutic supplements have importance in the market [20]. Some carotenoids such as astaxanthin, *β*-carotene, canthaxanthin, lutein, zeaxanthin, and lycopene are used commercially. *β*-Carotene is the pigment carotene, which is the major precursor of vitamin A that has many antioxidant and immune properties [21]. *β*-Carotene is trapping radical and considered as an unusual antioxidant [22]. These natural antioxidants when ingested with the diet prevent oxidative stress and induce synergisms [23]. This study was undertaken to investigate the therapeutic effect of the *D*. *salina* in TAA-induced HE. In order to give a better insight, we also examined the role of hyperammonemia, oxidative stress, or inflammation pathways by which *D*. *salina* exerts its therapeutic actions in brain via TLR4, HSP-25, and IGF-1 regulation.

## 2. Material and Methods

### 2.1. Cultivation of *D*. *salina* in the Photobioreactor

Algal species (*D*. *salina*) isolated from a salt pond in Al-Fayoum is grown by using Bold nutrient media containing sodium chloride with a concentration of 100 g/L for algal isolation and purification [24]. After growing *D*. *salina* for 10 days under lab conditions, it was then transferred to a vertical photobioreactor with a capacity of 4000 L. Reservoir (1000 L) tank associated pipework proprietary inline pigging systems. Also, 10 L basket centrifuge for harvesting was connected to the system. Alga Connect Data Acquisition System was used for online measurements. Tap water was used for the cultivation of algae in the photobioreactor (PBR). Water was sterilized using hypochlorite, and after that, sodium thiosulphate was added to remove the excess hypochlorite. The chlorine test was performed to ensure no residual chlorine is present. The nutrient solution of Bold was used for growing *D*. *salina*. One milliliter per liter of micronutrient solution was added to the culture medium. To ensure the purity of the culture, samples are taken regularly and examined microscopically. Carbon dioxide was injected into the culture as a carbon source. The culture is left to grow until the biomass reached the maximum (2–2.5 g/L). Algal biomass is harvested using the basket centrifuge at 2000 rpm, washed twice with tab water, and dried in the sun dryer where the temperature reached approximately 45°C and then grounded into a homogeneous fine powder.

### 2.2. Chemicals and Kits

Thioacetamide was purchased from Sigma-Aldrich Co., USA. Aspartate aminotransferase (AST), alanine aminotransferase (ALT), ammonia, reduced glutathione (GSH), and malondialdehyde (MDA) were purchased from Biodiagnostic, Cairo, Egypt. Toll-like receptor 4 (TLR4), heat shock protein (HSP-25), and insulin growth factor-1 (IGF-1) were purchased from NOVA, Beijing, China.

### 2.3. Animals

Forty male Wistar albino rats weighing 120–150 g were obtained from the animal house of the National Research Centre (Dokki, Cairo, Egypt) and were kept in standard polypropylene cages under standard environmental conditions with equal light-dark cycles. Rats were adapted for 1 week and were fed rat normal pellet diet and water ad libitum, before the beginning of the experiment. This experiment was carried out in accordance with the Ethics Committee of the National Research Centre, Egypt, and followed the National Institutes of Health Guide Recommendations Care and Use of Laboratory Animals (Publication No. 85-23, revised 1985).

### 2.4. Experimental Design

Rats were divided into 4 groups; the first group was injected with saline (2.5 ml/kg; i.p.) for three successive days and received distilled water (5 ml/kg; p.o.) for 7 days to be considered as a normal, while the second group injected with TAA (200 mg/kg; i.p.) for three successive days to induce HE and served as TAA control [25]. The third and fourth groups were administered *D*. *salina* (100 and 200 mg/kg; p.o.), respectively [26, 27], after TAA injection for 7 days.

### 2.5. Behavioral Test (Rotarod Test)

Motor coordination of rats was assessed using an accelerating rotarod (Model No. 7750; Ugo Basile), according to the procedure described by [28]. Rats were given three training sessions on three successive days. All rats were trained on the rotarod apparatus at a fixed speed of 4 rotations per minute (rpm) to reach a stable performance, before starting treatment with *D*. *salina*. On the fourth day, the rats were placed on the testing rod and the speed of the rotarod started at 4 rpm and then increased gradually to reach 40 rpm over 300 s. The basal falling latency time for each rat was recorded using a cutoff limit of 300 s. After the last administration of *D*. *salina*, each rat was then replaced on the accelerating rotarod apparatus for 300 s test sessions and the final falling latency time was recorded [29].

### 2.6. Serum Biochemical Analysis for Liver Enzyme and Ammonia

At the end of the experiment, rats were anesthetized with pentobarbital sodium and blood samples were withdrawn from the retroorbital venous plexus. Collected blood samples were allowed to stand for 10 min at room temperature then centrifuged at 4°C using a cooling centrifuge (Laborezentrifugen, 2k15, Sigma, Germany) at 3000 rpm for 10 min [30]. Sera were separated for assessment of AST, ALT, and ammonia [31].

### 2.7. Preparation of Tissue Homogenate

The brain was then excised, washed with saline, and placed in ice-cold phosphate buffer (pH 7.4) using a tissue homogenizer (MPW−120, Bit-Lab Medical instruments, Poland) to prepare 20% homogenate. Homogenized tissues were centrifuged at 4000 rpm/min for 10 min at 4°C using a cooling centrifuge (Laboratory Centrifuge, 2K15, Sigma Co., Germany). The supernatant was collected and stored at −80°C [32] and then used for the estimation of brain contents of TLR4, HSP-25, and IGF-1.

### 2.8. Assessment of Oxidative Stress

Lipid peroxides were estimated as thiobarbituric acid-reactive substances (TBARS) [33]. The absorbance of the resulting pink color was measured at 532 nm. The serum level of reduced glutathione (GSH) was determined. Both protein and nonprotein thiol (–SH) groups (mainly GSH) react with Ellman's reagent [5,5-dithiobis (2-nitrobenzoic acid)] to form a stable yellow color of 5-mercapto-2-nitrobenzoic acid, which can be measured colorimetrically at 412 nm [34].

### 2.9. Assessment of Brain Contents of TLR4, HSP-25, and IGF-1

Brain contents of TLR4, HSP-25, and IGF-1 were determined using ELISA (Enzyme-Linked Immunosorbent Assay) kit. We followed the manufacturer's instructions of the NOVA kit, Beijing, China, for calculating the results. Standards and samples were pipetted into wells with immobilized antibodies specific for rat TLR4, HSP-25, and IGF-1 and then were incubated. After incubation and washing, biotinylated antirat TLR4, HSP-25, and IGF-1 antibodies were added. Any unbound substances were washed away; horseradish peroxidase-conjugated streptavidin was pipetted into the wells, which were washed once again. TMB (tetramethylbenzidine) substrate solution was added to the wells; color developed proportionally to the amount of TLR4, HSP-25, and IGF-1 bound. Color development was discontinued (Stop Solution), and its intensity was measured at 450 nm.

### 2.10. Statistical Analysis

All the values are presented as means ± standard error of the means (SE). Data were evaluated by one-way analysis of variance followed by Tukey's multiple comparisons test. The Graph pad Prism software, version 5 (Inc., San Diego, USA) was used to carry out these statistical tests. The difference was considered significant when *p* < 0.05.

### 2.11. Histopathological Examination of Brain Tissue

Brain tissues were harvested from the different groups and fixed in 10% neutral buffered formalin. The brain tissues were processed to obtain 4 *μ*m paraffin-embedded sections then stained with hematoxylin and eosin stain (H&E). To determine an appropriate scoring system for brain tissue changes, the scores were derived semiquantitatively using light microscopy.

## 3. Results

### 3.1. Effect of *D*. *salina* on the Behavioral Test (Rotarod Test)

**Figure 1:**
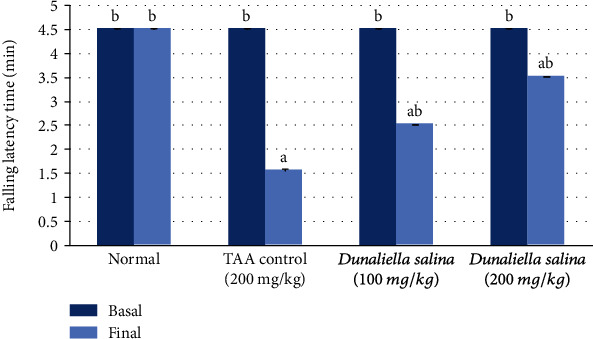
Effects of *D*. *salina* powder on the behavioral test (rotarod test). Data are presented as the mean ± SE of *n* = 10 for each group. Statistical analysis was carried out by one-way analysis of variance followed by Tukey's multiple comparisons test. ^a^Statistically significant from normal group. ^b^Statistically significant from the TAA control at *p* < 0.05.

The induction of HE by TAA (200 mg/kg; i.p.) for three alternative days revealed a significant reduction in the final falling downtime by 65% when compared to the normal. Oral administration of *D*. *salina* (100 and 200 mg/kg) for 7 consecutive days significantly elevated final falling downtime by 61% and 125%, respectively, as compared to the TAA control (Figure 1).

### 3.2. Effect of *D*. *salina* on Liver Enzymes and Serum Ammonia

TAA injection resulted in a significant rise in serum ALT, AST, and ammonia levels by 83%, 72%, and 134%, respectively, when compared to the normal. Posttreatment with *D*. *salina* (100 and 200 mg/kg) for 7 consecutive days decreased serum levels of ALT by 28% and 44% and serum AST by 10% and 21%, as well as ammonia by 19% and 39%, respectively, in comparison to TAA control. Also, treatment with *D*. *salina* (200 mg/kg) returned serum ALT level to the normal value (Table 1).

### 3.3. Effect of *D*. *salina* on Oxidative Stress

As depicted in Table 2, HE induced by TAA significantly increased MDA serum level by 99% and decreased GSH serum level by 58% when compared to normal. Posttreatment with *D*. *salina* (100 and 200 mg/kg) significantly ameliorated serum MDA level by 20% and 35%, respectively, and elevated serum GSH level by 94% and 112%, respectively, when compared to TAA control. Moreover, treatment with *D*. *salina* (200 mg/kg) restored the MDA level to normal value.

### 3.4. Effect of *D*. *salina* on TLR4, HSP-25, and IGF-1

**Figure 2:**
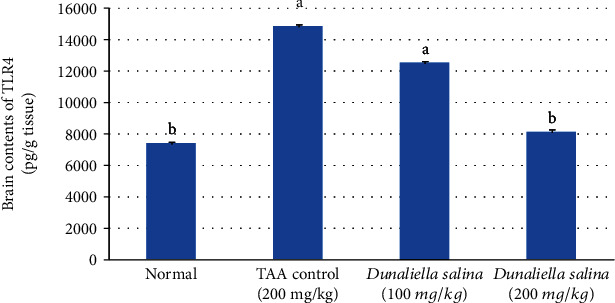
Effects of *D*. *salina* powder on hepatic contents of TLR4. Data are presented as the mean ± SE of *n* = 10 for each group. Statistical analysis was carried out by one-way analysis of variance followed by Tukey's multiple comparisons test. ^a^Statistically significant from normal group. ^b^Statistically significant from the TAA control at *p* < 0.05.

Brain TLR4 content was significantly elevated in the TAA control by 1 fold when compared to normal. Oral treatment with *D*. *salina* (200 mg/kg) significantly reduced TLR4 content by 45% and returned it to normal value, while *D*. *salina* (100 mg/kg) did not reduce it when compared to the TAA control (Figure 2).

**Figure 3:**
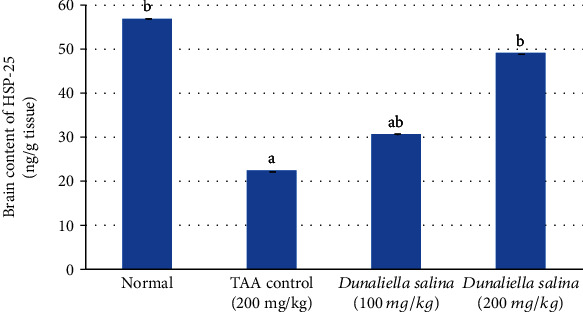
Effects of *D*. *salina* powder on hepatic contents of HSP-2. Data are presented as the mean ± SE of *n* = 10 for each group. Statistical analysis was carried out by one-way analysis of variance followed by Tukey's multiple comparisons test. ^a^Statistically significant from normal group. ^b^Statistically significant from the TAA control at *p* < 0.05.

TAA injection significantly decreased brain HSP-25 content by 61% when compared to normal. *D*. *salina* (100 and 200 mg/kg) posttreatment increased HSP-25 brain content by 38% and 121%, respectively, when compared to the TAA control. Moreover, treatment with *D*. *salina* (200 mg/kg) restored HSP-25 to the normal value (Figure 3).

**Figure 4:**
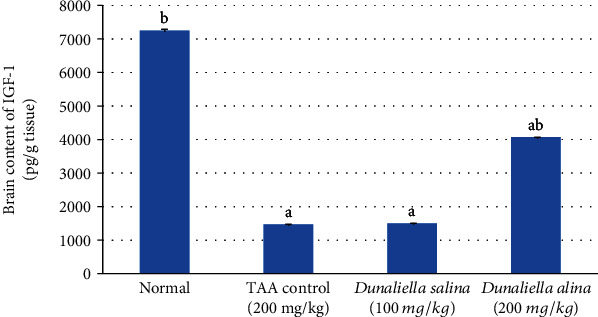
Effects of *D*. *salina* powder on hepatic contents of IGF-1. Data are presented as the mean ± SE of *n* = 10 for each group. Statistical analysis was carried out by one-way analysis of variance followed by Tukey's multiple comparisons test. ^a^Statistically significant from normal group. ^b^Statistically significant from the TAA control at *p* < 0.05.

Induction of HE produced a decrease in brain content of IGF-1 by 80% when compared to normal, while the administration of *D*. *salina* at a dose of 200 mg/kg increased it by 1.79 folds. However, the administration of *D*. *salina* at a dose of 100 mg/kg did not change it, when compared to the TAA control (Figure 4).

### 3.5. Histopathological Findings

#### 3.5.1. Effects of *D*. *salina* Powder on the Cerebral Cortex

**Figure 5:**
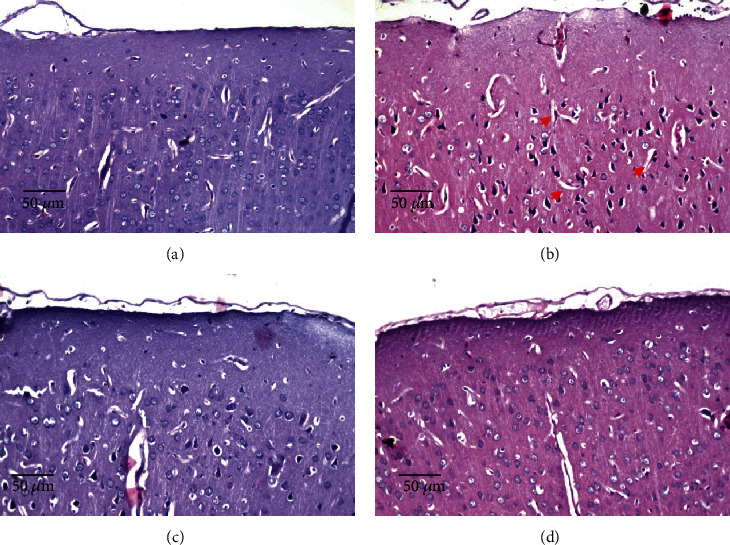
Effects of *D. salina* powder on cerebral cortex. (a) Normal showed no histopathological alteration and the normal histological structure of the neurons. (b) TAA control showed nuclear pyknosis and degeneration in most of the neurons (red arrow). (c) A low dose of *D*. *salina* showed no histopathological alteration. (e) A high dose of *D*. *salina* showed no histopathological alteration (H&E stain, ×200 scale bar 50 *μ*m).

Normal group showed no histopathological alteration and the normal histological structure of the neurons was recorded in cerebral cortex. TAA control showed nuclear pyknosis and degeneration in most of the neurons (red arrow). Both doses of *D*. *salina* showed no histopathological alteration (Figure 5).

#### 3.5.2. Effects of *D*. *salina* Powder on the Hippocampus

**Figure 6:**
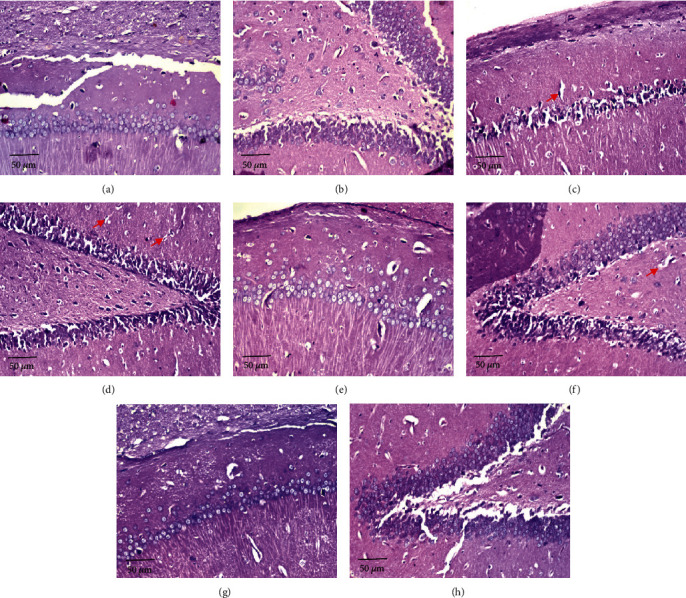
Effects of *D. salina* powder on the hippocampus. (a) Normal showed no histopathological alteration and the normal histological structure of the neurons was recorded in the subiculum. (b) Normal showed no histopathological alteration and the normal histological structure of the neurons was recorded in fascia dentata and hilus. (c) TAA control showed nuclear pyknosis and degeneration in most of the neurons in the subiculum (red arrow). (d) TAA group showed nuclear pyknosis and degeneration in most of the neurons in fascia dentata and hilus (red arrow). (e) A low dose of *D*. *salina* showed no histopathological alteration in the subiculum. (f) A low dose of *D*. *salina* showed nuclear pyknosis and degeneration in some neurons in fascia dentata and hilus (red arrow). (g) High dose of *D*. *salina* showed no histopathological alteration as recorded in the subiculum. (h) A high dose of *D*. *salina* showed no histopathological alteration in fascia dentata and hilus (H&E stain, ×200 scale bar 50 *μ*m).

Normal group showed no histopathological alteration, and the normal histological structure of the neurons was recorded in the subiculum and fascia dentata and hilus. TAA control showed nuclear pyknosis and degeneration in most of the neurons in the subiculum and the subiculum, fascia dentata, and hilus (red arrow). A low dose of *D*. *salina* showed no histopathological alteration in the subiculum. A low dose of *D*. *salina* showed nuclear pyknosis and degeneration in some neurons in the subiculum, fascia dentata, and hilus (red arrow). High dose of *D*. *salina* showed no histopathological alteration as recorded in the subiculum and fascia dentata and hilus (Figure 6).

#### 3.5.3. Effects of *D*. *salina* Powder on the Striatum

**Figure 7:**
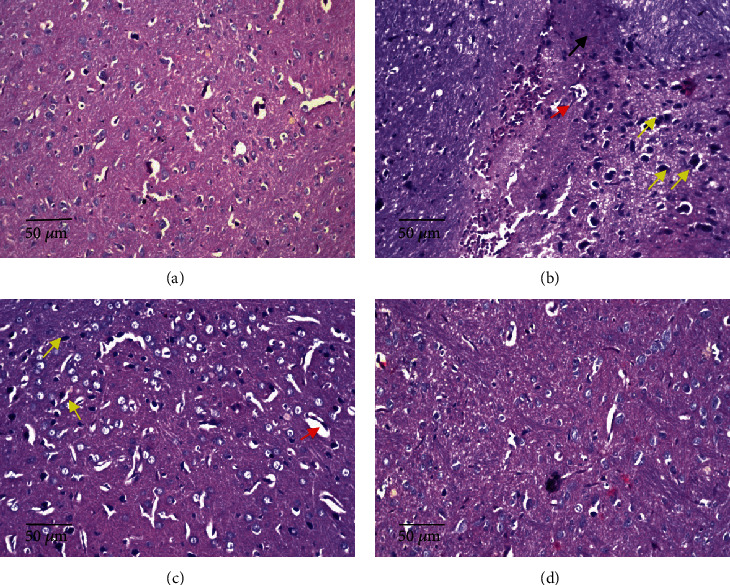
Effects of *D. salina* powder on striatum: (a) normal showed no histopathological alteration and the normal histological structure of the neurons was recorded. (b) TAA control showed diffuse gliosis (yellow arrow) and focal hemorrhage (black arrow) in between the nuclear pyknosis and degenerated neurons (red arrow). (c) A low dose of *D*. *salina* showed diffuse gliosis (yellow arrow) was noticed in between the nuclear pyknotic nuclear and degenerated neurons and another intact one (red arrow). (d) A high dose of *D*. *salina* showed no histopathological alteration (H&E stain, ×200 scale bar 50 *μ*m).

Normal showed no histopathological alteration and the normal histological structure of the neurons was recorded in striatum. TAA control showed diffuse gliosis (yellow arrow) and focal hemorrhage (black arrow) in between the nuclear pyknosis and degenerated neurons (red arrow). A low dose of *D*. *salina* showed diffuse gliosis (yellow arrow) was noticed in between the nuclear pyknotic nuclear and degenerated neurons and another intact one (red arrow). A high dose of *D*. *salina* showed no histopathological alteration (Figure 7). The severity of alteration in the brain was blindly scored microscopically, and scores are presented in Table 3.

## 4. Discussion

Hepatic encephalopathy (HE) is a syndrome arising from acute or chronic liver diseases and is considered as a neuropsychiatric complication [35]. In the present work, the results showed that thioacetamide (TAA) induced a state of liver dysfunction linked with brain affection. Animals that injected with TAA showed a significant elevation in AST and ALT activities with a concomitant rise in ammonia level. TAA is a hepatotoxin inducing hepatic failure and elevating serum levels of AST and ALT [36] which expressed in hepatocyte cytoplasm. They are entering the bloodstream after hepatocyte injury as the cell membrane permeability increases. Their elevation reflects the degree of hepatocyte damage [37]. There was a significant increase; also, in the blood, ammonia level was observed in the TAA control, compared to the normal, in a previous study [38]. This hyperammonemia is due to liver insufficient detoxification and the reduced urinary loss of ammonia leading to direct ammonia provoked neurotoxicity [39]. On the other hand, rats have given both doses of *D*. *salina* for 7 consecutive days significantly decreased serum activities of ALT and AST, as well as ammonia level, compared to TAA control. These results indicate the beneficial role of *D*. *salina* in restoring liver integrity. In line with these results, our previous work showed that the administration of *D*. *salina* for 1 month decreased serum activities of ALT and AST and has an antifibrotic effect against TAA-induced liver fibrosis [40]. Moreover, treatment with *D*. *salina* preserved hepatocyte integrity [40].

Hyperammonemia-induced neuroinflammation activated cerebellar astrocytes and microglia and produced cerebellum motor coordination dysfunction [41]. In HE patients, there was subclinical motor slowing and impaired visuoconstructive ability, visual perception, and mild cognitive dysfunction [42]. In our study, TAA injection exhibited a significant decline in the final falling downtime in the rotarod test due to motor slowing and dysfunction compared to normal. This result is in line with a previous study [43]. However, oral administration of both doses of *D*. *salina* significantly increased the final falling downtime as compared to the TAA control as a result of decreasing ammonia brain level and suggesting that *D*. *salina* restored motor and cognitive functions, especially high dose.

**Figure 8:**
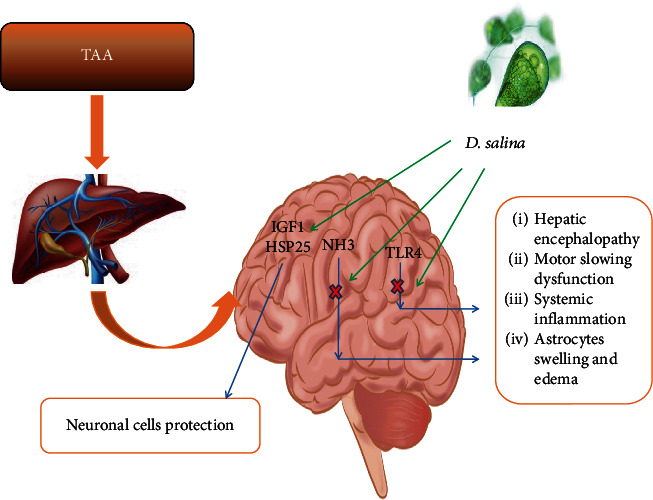
Effects of *D*. *salina* powder on hepatic encephalopathy through hyperammonemia/TLR4 pathway.

A close correlation between ammonia level elevation and toll-like receptor (TLR4) upregulation is found in astrocytes and endothelial cells (ECs), under neuroinflammatory conditions, and worsen HE [44, 45]. TLR4 protein is one of the common factors responsible for inflammatory mediators release from brain ECs and microglia after ammonia exposure resulting in astrocyte swelling. TLR4 stimulates nuclear factor-kappa B (NF-*κ*B) that releases proinflammatory cytokines tumour necrosis factor-alpha (TNF-*α*), interleukin 1 beta (IL-1*β*), and interleukin 6 (IL-6) which is involved in immune responses [46, 47]. Together with ammonia, TNF-*α* upregulation in astrocytes affects neuronal survival, learning, and memory [48]. In the present work, TAA induced liver dysfunction and hyperammonemia that associated with enhanced expression of TLR4 brain content as compared to normal rats (Figure 8). In HE, TAA caused brain TLR4 elevation in mice [49], while the oral administration of the high dose of *D*. *salina*, only, was significantly reduced brain TLR4 content as compared to its normal value, suggesting antiinflammatory effects of its components; *β*-carotene and zeaxanthin.

Increased ammonia, also, produced astrocyte swelling, reactive nitrogen, and oxygen radical formation, lipid peroxidation elevation [50], and reduction of the activity of antioxidant enzymes in the brain [51, 52] and amplifies the neuronal derangements [53]. In the present work, TAA-induced hyperammonemia reduced glutathione (GSH) and elevated malondialdehyde (MDA) serum levels as compared to control rats. On the other hand, oral treatment especially with a high dose of *D*. *salina* elevated serum GSH level and returned MDA level to its normal value. These results may suggest that *D*. *salina* is a potent anti-inflammatory and antioxidant agent in the nervous system that suppresses inflammatory and oxidative stress pathways through its carotenoid contents such as zeaxanthin and *β*-carotene, treating HE. Indeed, recent studies explored that carotenoids inhibit NF-*κ*B activity [54] regulating inflammation-related and oxidative stress genes in neurodegenerative diseases [55]. Also, marine carotenoids have antioxidant properties by activation of the antioxidant network (GSH and catalase) [56] and scavenging reactive oxygen species (ROS) [57]. *D*. *salina*, in another study, regulates oxidative stress and protects the liver from fibrosis [16].

Increased cellular stress induces the expression of heat shock proteins (HSPs) affecting neurons' response [58]. HSP72 and HSP 25/27 play a role in cellular protection [59]. HSP-25 downregulates 6-hydroxydopamine-induced cytochrome c release and apoptosis and protects complex I activity during oxidative stress. Thus, elevated HSP-25 expression is a defense mechanism for neuronal cells under stress conditions, via its antioxidant properties [60]. Increased HSP expression is linked to attenuation of the proinflammatory cytokines as well as oxidative stress [61]. Regarding, TAA injection significantly induced brain oxidative stress and inflammation associated with decreased brain HSP-25 content when compared to normal rats. The p38 mitogen-activated protein kinase (MAPK) isoform is upregulated in response to oxidative stress or inflammation which in turn modulates antioxidants, inflammatory mediators, and survival gene expression [62]. p38a controls the expression of HSP-25 preventing oxidative stress. Restoration of HSP-25 expression plays an important role in TAA-induced ROS accumulation and fibrogenesis [63]. In another study, TAA injection showed an inverse correlation between HSP-25 expression and ROS release in mice [64]. Both doses of *D*. *salina* posttreatment enhanced HSP-25 brain content when compared to the TAA control exerting defense mechanism for neuronal cells against oxidative damage through the potant antioxidant activity of *β*-carotene and zeaxanthin, especially high dose.

Neurodegeneration development, also, is influenced by insulin-like growth factor-1 (IGF-1) disruption. IGF-1 plays a vital role in the regulation of growth and metabolism [65]. IGF-1 inhibits brain oxidative insults [66] and protects neurons against the diverse effect of oxidative stress [67, 68]. In our study, the induction of HE produced a decrease in brain content of IGF-1 with nuclear pyknosis and degeneration in most of the neurons, when compared to normal rats. In an agreement with our study, HE induced by azoxymethane suppressed IGF-1 expression [69] and produced nuclear pyknosis and neuron degeneration [70]. This study, for the first time, showed the effect of *D*. *salina* on IGF-1 in brain tissue. Administration of a high dose of *D*. *salina* caused a significant increase in brain content of IGF-1, when compared to the TAA control and inhibited nuclear pyknosis and neuron degeneration induced by TAA, indicating its therapeutic role in HE.

## 5. Conclusion

*D*. *salina* has antioxidative and anti-inflammatory activities in the brain. It controls liver function, ammonia, and behavioral changes. Also, *D*. *salina* regulates lipid peroxidation, an antioxidant enzyme, TLR4, HSP-25, and IGF-1, exhibiting neurotherapeutic activity, so further works are needed to examine its clinical benefits in brain pathologies associated with oxidative stress and inflammation.

## Figures and Tables

**Table 1:** Effects of *D*. *salina* powder on serum hepatic functions biomarkers and ammonia.

	Normal	TAA control (200 mg/kg)	*Dunaliella salina* (100 mg/kg)	*Dunaliella salina* (200 mg/kg)
ALT (U/L)	40.35 ± 0.36^b^	73.98 ± 0.88^a^	53.10 ± 0.63^ab^	41.42 ± 0.23^b^
% of TAA		100%	72%	56%
AST (U/L)	63.22 ± 0.42^b^	108.68 ± 0.68^a^	98.20 ± 0.31^ab^	85.85 ± 0.54^ab^
% of TAA		100%	90%	79%
Ammonia (*μ*mol/L)	135.80 ± 0.26^b^	317.80 ± 4.04^a^	257.80 ± 3.39^ab^	195.20 ± 0.33^ab^
% of TAA		100%	81%	61%

Data are presented as the mean ± SE of *n* = 10 for each group and presented as % of the TAA control. Statistical analysis was carried out by one-way analysis of variance followed by Tukey's multiple comparisons test. ^a^Statistically significant from normal group. ^b^Statistically significant from the TAA control at *p* < 0.05.

**Table 2:** Effects of *D*. *salina* powder on serum oxidative stress biomarkers.

	Normal	TAA control (200 mg/kg)	*Dunaliella salina* (100 mg/kg)	*Dunaliella salina* (200 mg/kg)
GSH (mg/dl)	15.56 ± 0.03^b^	6.53 ± 0.06^a^	12.67 ± 0.08^ab^	13.87 ± 0.03^ab^
% of TAA		100%	194%	212%
MDA (nmol/ml)	9.74 ± 0.07^b^	19.36 ± 0.29^a^	15.43 ± 0.07^ab^	12.56 ± 0.10^b^
% of TAA		100%	80%	65%

Data are presented as the mean ± SE of *n* = 10 for each group and presented as % of the TAA control. Statistical analysis was carried out by one-way analysis of variance followed by Tukey's multiple comparisons test. ^a^Statistically significant from normal group. ^b^Statistically significant from the TAA control at *p* < 0.05.

**Table 3:** Effects of *D*. *salina* powder on histopathological scoring in brain tissue.

		Normal	TAA control (200 mg/kg)	*Dunaliella salina* (100 mg/kg)	*Dunaliella salina* (200 mg/kg)
Cerebral cortex	Nuclear pyknosis and neuronal degeneration	**--**	**+++**	**--**	**--**
Hippocampus	Nuclear pyknosis and neuronal degeneration	**--**	**++**	**+**	**--**
Striatum	Focal hemorrhage	**--**	**++**	**--**	**--**
	Nuclear pyknosis and neuronal degeneration	**--**	**+++**	**++**	**--**
	Gliosis	**_**	**++**	**+**	**--**

+++: sever histopatholocical alteration. ++: moderate histopatholocical alteration. +: mild histopatholocical alteration. --: nil histopatholocical alteration.

## Data Availability

The data used to support the findings of this study are available from the corresponding author upon request.
